# Efficacy of Laser Therapy in Patients With Atopic Dermatitis: A Scoping Review

**DOI:** 10.7759/cureus.105694

**Published:** 2026-03-23

**Authors:** Calista Persson, Leili Niu, Chaimae Oualid, Amnah Azeem, Jad Kawouk, Michelle Demory

**Affiliations:** 1 Dr. Kiran C. Patel College of Osteopathic Medicine, Nova Southeastern University, Davie, USA; 2 Division of Immunology, Dr. Kiran C. Patel College of Allopathic Medicine, Nova Southeastern University, Davie, USA

**Keywords:** 308-nm excimer laser, atopic dermatitis (ad), dermatology cosmetology, eczema, filaggrin, medical and cosmetic dermatology, nanosecond-domain nd:yag laser, narrow-band ultraviolet b, phototherapy efficacy, phototherapy modalities

## Abstract

Atopic dermatitis (AD), or eczema, is a chronic inflammatory skin condition marked by itching, redness, and irritation. Traditional treatments like moisturizers, topical steroids, and antihistamines offer varied results and often cause side effects. As a result, interest has grown in alternative therapies, including narrow-band ultraviolet B (NB-UVB) phototherapy and laser-based treatments.

This scoping review evaluates the efficacy and relevance of laser therapies in managing AD.

A literature search was conducted in September 2023 using EMBASE, Ovid MEDLINE, and Web of Science. Eligible studies included original, English-language research published between 2013 and 2023 that focused on phototherapy for AD. Studies were excluded if they involved non-human subjects, were reviews or opinion pieces, lacked full-text access, or were policy/guideline documents. Twenty-seven studies met the inclusion criteria.

Two laser modalities demonstrated notable potential for treating AD. The 308 nm excimer laser was shown to reduce scratching behavior in dermatitis mouse models across three studies. Picosecond- and nanosecond-domain Nd:YAG lasers improved the penetration of topical peptides and accelerated skin barrier recovery. However, no studies directly examined their effects in human or animal models of AD. Additionally, none of the reviewed studies assessed the efficacy of these laser therapies across different patient populations, revealing a significant gap in the literature. Although molecular research has identified potential therapeutic targets in AD, no direct links between these targets and laser treatment have been established.

Laser therapies, particularly the 308 nm excimer and Nd:YAG lasers, show promise for managing AD. However, further research is needed to evaluate their long-term effectiveness, impact on symptoms, and overall benefits for diverse patient populations.

## Introduction and background

Atopic dermatitis (AD) is a common chronic inflammatory skin disease affecting approximately 15-20% of children and 1-10% of adults worldwide [1-3]. Although its name suggests an atopic origin, AD can arise independently or in conjunction with other allergic disorders such as asthma and allergic rhinitis, particularly in individuals with a family history of atopy [3,4]. The condition is characterized by recurrent episodes of pruritus (often severe and worse at night), along with dry, sensitive skin; erythematous to brownish-gray patches; excoriated or oozing papules; and thickened, lichenified plaques in chronic stages [1,5,6]. Cracking, scaling, and skin barrier dysfunction further predispose patients to secondary bacterial or viral infections. Though AD typically manifests during infancy or early childhood, about 25% of cases persist into adulthood, and new-onset disease in adults is also increasingly recognized [3,4]. The pathogenesis involves a multifactorial interplay of genetic mutations (e.g., filaggrin gene defects), immune dysregulation, environmental triggers, and epidermal barrier dysfunction. While many children achieve remission by adolescence, studies indicate that only around 60% experience complete resolution by early adulthood, and up to 40% continue to have intermittent or chronic symptoms [3,6].

Typical treatment for AD

The treatment and management of AD, commonly known as eczema, follow a stepwise and individualized approach based on disease severity, aiming to relieve symptoms, reduce inflammation, prevent flare-ups, and improve quality of life [2,7,8]. First-line therapy focuses on restoring the skin barrier and reducing dryness -- regular and liberal use of emollients and moisturizers is essential to prevent transepidermal water loss and reduce flare frequency [1,3]. Patients are also advised to avoid known irritants and triggers (such as harsh soaps, detergents, allergens, and extreme temperatures), use gentle skin cleansers, and maintain optimal skin hydration [2,3].

For acute flares, topical corticosteroids (TCS) and calcineurin inhibitors are commonly prescribed to reduce inflammation. In some cases, oral antihistamines may help relieve severe pruritus, especially at night, though their efficacy varies [6,9]. In patients with moderate-to-severe AD unresponsive to topical agents, second-line therapies include phototherapy (e.g., narrowband UVB) and systemic immunomodulators such as cyclosporine, methotrexate, or azathioprine [10-13]. More recently, biologic agents like dupilumab, an IL-4 receptor antagonist, have demonstrated significant benefit and are FDA-approved for moderate-to-severe cases [4,6,8].

Lifestyle modifications play a critical adjunctive role, particularly in pediatric patients. These include wearing soft, breathable clothing, avoiding overheating, using humidifiers in dry environments, establishing consistent skincare routines, and adopting stress-reducing practices such as mindfulness or behavioral therapy [2-4]. While AD remains a chronic condition with recurrent flares and remissions, many individuals achieve effective long-term symptom control and extended periods of clear skin through proactive management strategies [9].

Phototherapy use for AD

Laser therapy was considered for the treatment of patients with AD. In some cases, the standard of care, which included TCS, moisturizers, and antihistamines, provided insufficient relief or led to long-term side effects in certain patients [9,13]. Alternative treatment modalities, such as laser therapy, were therefore investigated.

Phototherapy options that were tried included narrow-band ultraviolet B (NB-UVB) and excimer laser. NB-UVB included exposing the affected skin to NB-UVB light [14]. The specific wavelength used in NB-UVB has been shown to have anti-inflammatory effects, immune suppression, and an improvement in skin barrier function [15,16]. It was generally delivered at the medical facility over several weeks.

The excimer laser produces high-intensity UV light at a specific wavelength (usually 308 nm) to target affected lesions with minimal damage to surrounding normal skin. It was often used for focal, recalcitrant plaques. Excimer treatment sessions were usually shorter in duration and fewer in number than those of conventional NB-UVB therapy [16-19].

While laser therapy offers a valuable alternative or adjunct for some patients, its long-term safety, durability of response, and optimal patient selection remain areas of ongoing research. Clinical outcomes can be inconsistent, with varying degrees of improvement noted across different populations and disease phenotypes [20-23].

Gaps in the literature

Understanding the effectiveness of phototherapy modalities for AD is essential to guide clinical decision-making and support the development of alternative therapies. Although several studies have investigated treatments for AD, including moisturizers, TCS, antihistamines, and emerging laser-based interventions [19,23], a comprehensive mapping of the literature using a scoping review methodology has not previously been conducted.

In this review, we aimed to systematically review the literature on the use of laser and phototherapy modalities in AD, focusing on studies from the past decade to reflect current practice trends and evolving technologies. Unlike systematic reviews, which appraise study quality, scoping reviews offer a broad synthesis of the evidence landscape without formal quality evaluation [3]. Our approach included a narrative overview of AD pathophysiology, standard treatments, and phototherapy classifications. We then examined the reported outcomes of various laser therapies, such as NB-UVB and excimer laser, based on data from case series, cohort studies, and clinical trials.

By synthesizing these findings, we highlight both the potential applications of targeted phototherapy in AD management and the gaps in evidence concerning long-term efficacy, safety, and patient selection. This review serves as a foundation for future research and clinical guidance, emphasizing the need for individualized care and informed discussions between patients and dermatologists when considering laser-based treatment options.

## Review

Methods

Eligibility Criteria

An extensive literature search was conducted to evaluate the use of lasers as a treatment for AD. All articles had to be written in English, peer-reviewed, and published between 2015 and 2025 to be included. The primary disease focus of the articles had to be AD, and the primary treatment being analyzed had to be laser therapy. Articles of interest included experimental studies in adult human subjects and/or animal or cell models that can be applied to the human body. In addition, studies that provided treatment guidance or interventions for AD were included, as well as those studying the pathogenesis of the disease. While original research studies were of primary interest, a select few review studies were also included to evaluate the current literature best and place the findings in a clinical and mechanistic context. Articles that were not original research, including books, conference abstracts, non-peer-reviewed presentations, and other gray literature, were not included.

Information Sources and Search Strategy

Three primary databases were utilized: Ovid MEDLINE, EMBASE, and Web of Science. To ensure a comprehensive search on the study of focus, a set of keywords was included: “atopic dermatitis, eczema, laser therapy, phototherapy, chronic atopic dermatitis, acute atopic dermatitis, phototherapy efficacy, laser therapy efficacy, and eczema recurrence." All searches were conducted in October 2025.

**Figure 1:**
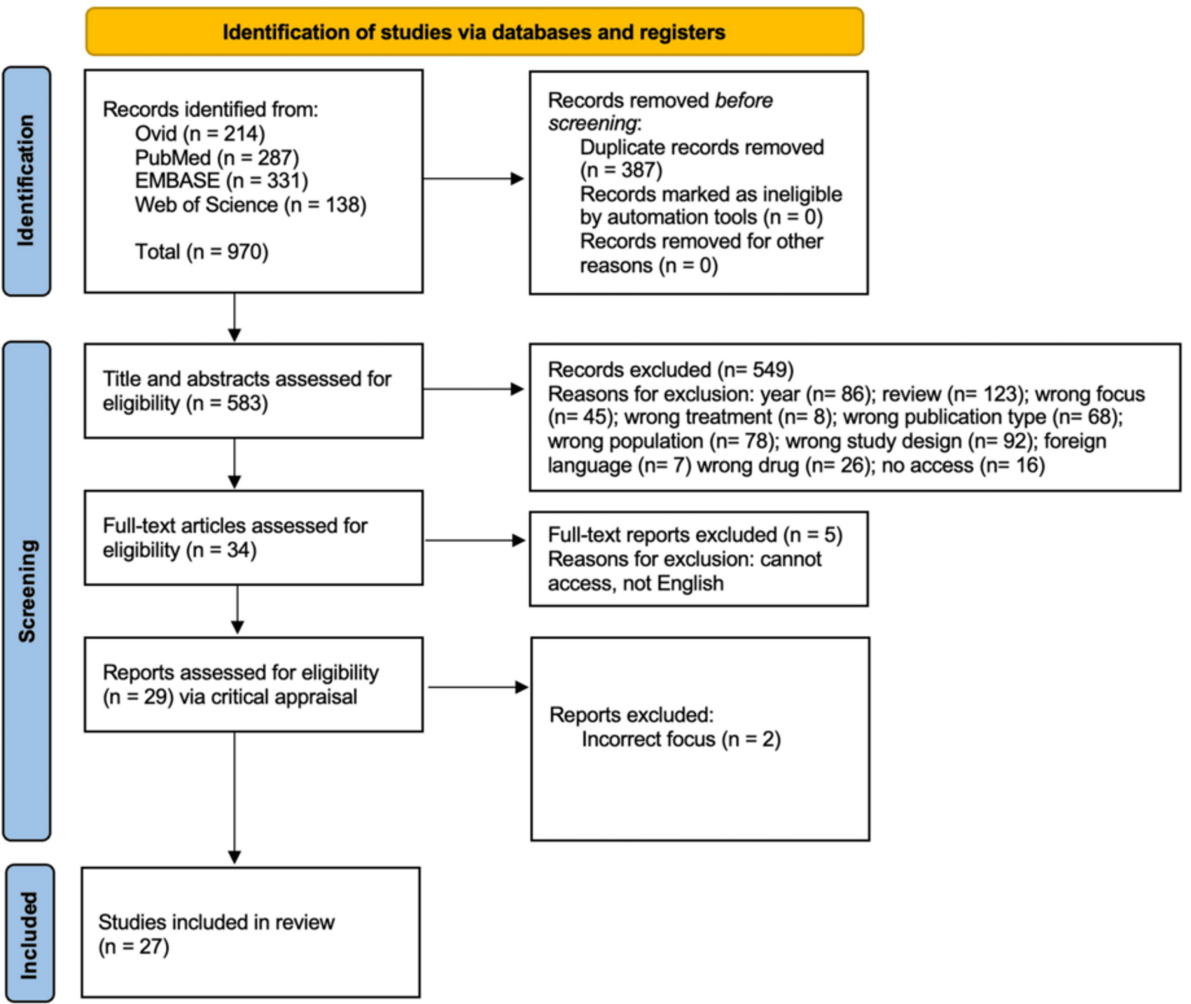
PRISMA Method Chart PRISMA = Preferred Reporting Items for Systematic Reviews and Meta-Analyses.

A total of 970 records were identified through database searches and uploaded into Rayyan (Rayyan Systems, Inc., Cambridge, MA) on October 13, 2025. After removal of 387 duplicates, 583 unique records remained for screening. Three independent reviewers screened titles and abstracts to assess inclusion criteria. During this stage, 549 records were excluded for not meeting eligibility criteria. Subsequently, 34 full-text articles were assessed in detail by two independent reviewers. Of these, five articles were excluded due to inaccessibility or language limitations. The remaining 29 studies underwent critical appraisal, with two studies excluded due to incorrect focus, resulting in 27 studies included in the final review. In cases of discordance, a third independent reviewer was consulted until consensus was achieved. The PRISMA flow diagram illustrates the study selection process [24] (see Figure 1).

Data Charting Process

A structured and comprehensive data charting form was developed to extract relevant information from the included sources of evidence. The form included fields aligned with the scoping review objectives, such as study objectives, design, setting, sample characteristics, main findings, limitations, and additional comments.

Items were selected for charting based on their relevance to the research questions and objectives. The data charting process involved multiple reviewers independently extracting information from selected sources. Calibration sessions were conducted among team members to ensure consistency in the interpretation and application of the charting form. Any discrepancies were resolved through discussion to achieve consensus. Data charting was conducted using Microsoft Excel (Microsoft, Redmond, WA).

The iterative nature of the charting process allowed for refinement of the data extraction tool. Modifications were made based on team discussions, and rationales for changes were documented. The finalized charting framework clearly defined each variable and ensured consistent data collection across studies.

A systematic approach was employed to obtain and verify extracted data. When necessary, corresponding authors or investigators were contacted to clarify ambiguities, confirm data points, or obtain additional information.

Data Items

Data items varied based on the focus of the scoping review and included both qualitative and quantitative variables. All extracted variables were explicitly defined to ensure consistency in interpretation. Data were independently extracted from selected studies by two or more reviewers using a standardized data extraction tool developed by the research team. The extracted data included specific details about the participants, concept, context, study methods, and key findings relevant to the review objectives. The reviewers utilized an Excel spreadsheet as the data extraction tool [25].

The data extraction tool was iteratively refined throughout the review process to ensure completeness and clarity. Any disagreements between reviewers were resolved through discussion or consultation with an additional reviewer. When appropriate, study authors were contacted to request missing or clarifying data.

Critical Appraisal of Individual Sources of Evidence

To ensure the quality and reliability of included evidence, critical appraisal was conducted when relevant to the scoping review objectives. The Joanna Briggs Institute (JBI) Critical Appraisal Tools [26] were used as the methodological framework for assessing study quality and potential bias. These tools were selected for their structured approach to evaluating various study designs, ensuring that included studies were methodologically sound and aligned with the review objectives.

The JBI Critical Appraisal Checklist is a standardized tool designed to assess methodological quality and potential bias in research studies. It evaluates key domains such as study design, participant selection, data collection methods, statistical analysis, and interpretation of results [26]. By systematically identifying strengths and limitations of individual studies, the checklist supports the inclusion of high-quality evidence and enhances the reliability of synthesized conclusions. Additionally, it facilitates the identification of potential sources of bias that may influence study outcomes [26-28].

In this review, the JBI Critical Appraisal Checklist was applied to selected studies on phototherapy for AD to ensure methodological rigor, minimize bias, and support evidence-based conclusions (see Table 1).

**Table 1:** JBI Critical Appraisal Checklist JBI = Joanna Briggs Institute.

Reference	Was the research question clearly defined?	Were the inclusion criteria appropriate?	Was the study design suitable for answering the research question?	Were study participants appropriately recruited?	Was data collection clearly described and systematically applied?	Were confounding factors identified and accounted for?	Was the statistical analysis appropriate and clearly reported?	Were results clearly presented and supported by data?	Was the conclusion based on the results?	Overall risk of bias (low/moderate/high)
Bieber T (2022) [1]	Yes	Yes	Yes	Yes	Yes	Partially	Yes	Yes	Yes	Low
Frazier W, Bhardwaj N (2020) [2]	Yes	Yes	Yes	Yes	Yes	Partially	Yes	Yes	Yes	Low
Golpour M et al. (2017) [3]	Yes	Yes	Yes	Yes	Yes	Partially	Yes	Yes	Yes	Low
Wollenberg A et al. (2018) [4]	Yes	Yes	Yes	Yes	Yes	Partially	Yes	Yes	Yes	Low
Renert-Yuval Y et al. (2021) [5]	Yes	Yes	Yes	Yes	Yes	Partially	Yes	Yes	Yes	Low
Mandlik DS, Mandlik SK (2021) [6]	Yes	Yes	Yes	Yes	Yes	Partially	Yes	Yes	Yes	Low
Puar N et al. (2021) [7]	Yes	Yes	Yes	Yes	Yes	Partially	Yes	Yes	Yes	Low
Sidbury R et al. (2014) [8]	Yes	Yes	Yes	Yes	Yes	Partially	Yes	Yes	Yes	Low
Kemény L et al. (2019) [9]	Yes	Yes	Yes	Yes	Yes	Partially	Yes	Yes	Yes	Low
Lee WR et al. (2018) [10]	Yes	Yes	Yes	Yes	Yes	Partially	Yes	Yes	Yes	Low
LaBrasca M et al. (2021) [11]	Yes	Yes	Yes	Yes	Yes	Partially	Yes	Yes	Yes	Low
Park KY et al. (2013) [12]	Yes	Yes	Yes	Yes	Yes	Partially	Yes	Yes	Yes	Low
Rodenbeck DL et al. (2016) [13]	Yes	Yes	Yes	Yes	Yes	Partially	Yes	Yes	Yes	Low
Kim YL et al. (2021) [14]	Yes	Yes	Yes	Yes	Yes	Partially	Yes	Yes	Yes	Low
Martirosyan D et al. (2022) [15]	Yes	Yes	Yes	Yes	Yes	Partially	Yes	Yes	Yes	Low
Bhatia BK et al. (2015) [16]	Yes	Yes	Yes	Yes	Yes	Partially	Yes	Yes	Yes	Low
Leguina-Ruzzi A et al. (2019) [17]	Yes	Yes	Yes	Yes	Yes	Partially	Yes	Yes	Yes	Low
Mehraban S, Feily A (2014) [18]	Yes	Yes	Yes	Yes	Yes	Partially	Yes	Yes	Yes	Low
Oh CT et al. (2016) [19]	Yes	Yes	Yes	Yes	Yes	Partially	Yes	Yes	Yes	Low
Choi SY et al. (2016) [20]	Yes	Yes	Yes	Yes	Yes	Partially	Yes	Yes	Yes	Low
Dhillon S, Lake E (2023) [21]	Yes	Yes	Yes	Yes	Yes	Partially	Yes	Yes	Yes	Low
Yamazaki K et al. (2021) [22]	Yes	Yes	Yes	Yes	Yes	Partially	Yes	Yes	Yes	Low
Chu H et al. (2017) [23]	Yes	Yes	Yes	Yes	Yes	Partially	Yes	Yes	Yes	Low
Dinish US et al. (2023) [29]	Yes	Yes	Yes	Yes	Yes	Partially	Yes	Yes	Yes	Low
Krupka-Olek M et al. (2022) [30]	Yes	Yes	Yes	Yes	Yes	Partially	Yes	Yes	Yes	Low
Kamo A et al. (2014) [31]	Yes	Yes	Yes	Yes	Yes	Partially	Yes	Yes	Yes	Low
Kurosaki Y et al. (2020) [32]	Yes	Yes	Yes	Yes	Yes	Partially	Yes	Yes	Yes	Low
Hagiyama M et al. (2013) [33]	Yes	Yes	Yes	Yes	Yes	Partially	Yes	Yes	Yes	Low
Kobiela A et al. (2023) [34]	Yes	Yes	Yes	Yes	Yes	Partially	Yes	Yes	Yes	Moderate
Liew WC et al. (2020) [35]	Yes	Yes	Yes	Yes	Yes	Partially	Yes	Yes	Yes	Moderate
Lu J et al. (2018) [36]	Yes	Yes	Yes	Yes	Yes	Partially	Yes	Yes	Yes	Moderate
Segaud J et al. (2022) [37]	Yes	Yes	Yes	Yes	Yes	Partially	Yes	Yes	Yes	Moderate
Luger T et al. (2021) [38]	Yes	Yes	Yes	Yes	Yes	Partially	Yes	Yes	Yes	Moderate
Luger T et al. (2020) [39]	Yes	Yes	Yes	Yes	Yes	Partially	Yes	Yes	Yes	Moderate
Szegedi K et al. (2015) [40]	Yes	Yes	Yes	Yes	Yes	Partially	Yes	Yes	Yes	Moderate
Try C et al. (2023) [41]	Yes	Yes	Yes	Yes	Yes	Partially	Yes	Yes	Yes	Moderate

Integration of Critical Appraisal Findings

The findings from the critical appraisal step had been integrated into the overall interpretation of the evidence [27,28]. Studies that demonstrated higher methodological rigor had been given greater weight in the synthesis of findings, while studies with a high risk of bias had been interpreted cautiously, with limitations explicitly acknowledged. The rationale for performing a critical appraisal had been clearly tied to the scoping review objectives, ensuring that the review mapped available evidence and assessed its quality and reliability. This methodological rigor had strengthened the interpretability and applicability of the review findings, providing a robust foundation for future research and clinical recommendations regarding phototherapy for AD.

Synthesis of the Results

The synthesis plan involved presenting the range of evidence in a narrative format, tables, and visual representations, including maps or diagrams. The synthesis had been guided by the research questions and objectives, providing a comprehensive overview of the efficacy of laser therapy in treating atopic dermatitis.

Results

A total of 27 studies had been identified using the study selection process illustrated in the PRISMA flowchart in Figure 1. Table 2 summarizes the characteristics of the studies included in this scoping review.

**Table 2:** Summary Table of Articles AD, atopic dermatitis; UVB, ultraviolet B; IL, interleukin; TSLP, thymic stromal lymphopoietin; TNF, tumor necrosis factor; IFN, interferon; SCORAD, Scoring Atopic Dermatitis Index; HDAC, histone deacetylase; FLG, filaggrin; JNK, c-Jun N-terminal kinase; PKC, protein kinase C; c-Src, cellular sarcoma kinase; ICAM, intercellular adhesion molecule.

Authors/Year	Aim of study	Study setting, participants' details	Methods including type of study and study design	Main findings	Limitations of the study
Frazier and Bhardwaj (2020) [2]	Objectives of the study were not explicitly outlined. However, the article discusses the characteristics, diagnostic criteria, and treatment options for atopic dermatitis without specifically detailing research objectives or goals.	Sampling methods: No sampling methods or research methodology are specified. Time frame: No specific time frame or duration for a research study is mentioned.	Type: There is no mention of a specific study design.	The text outlines treatment approaches and diagnostic criteria for atopic dermatitis. It covers recommended treatments, including emollients, topical corticosteroids, calcineurin inhibitors, phototherapy, anti-Staphylococcal antibiotics, and newer medications like crisaborole and dupilumab. Additionally, it highlights the lack of evidence supporting integrative medicine for this condition.	The study's limitations include a lack of discussion on novel and cost-prohibitive treatments like crisaborole and dupilumab, limited evidence supporting integrative medicine for atopic dermatitis, and a potential bias toward established treatments such as topical corticosteroids, which may overlook emerging therapies.
Wollenberg et al. (2018) [4]	The study aimed to establish consensus-based European guidelines for the treatment of atopic eczema (atopic dermatitis) in both adults and children. It incorporated input from diverse disciplines including physicians and patients, integrating available evidence from various sources such as other guidelines, systematic reviews, and published studies. The guideline is divided into parts, focusing on different aspects of the condition, providing comprehensive recommendations for management and treatment strategies.	Sampling methods and time frame: Information regarding the specific sampling method or time frame is not explicitly provided in the text.	Type: Consensus-based guideline development. The study represents a collaborative, joint interdisciplinary European project that includes physicians from various relevant disciplines and incorporates patient perspectives.	The study outlines key strategies for managing atopic eczema, emphasizing individualized treatment for short-term flares and long-term control.	Authors did not limit the selection of evidence to a specific study design. The trials evaluating the efficacy of emollients on AD were limited by their short durations. There is limited evidence-based data for the antipruritic effect of AH (H1 antagonists) in AE in general, and the effect of both first- and second-generation AH on pruritus in patients suffering from AE is very limited.
Lee et al. (2018) [10]	The study aimed to determine whether the fractional laser could enhance peptide and siRNA penetration in diseased skin, particularly in models of psoriasis-like and atopic dermatitis (AD)-like skin. Additionally, it sought to evaluate the effect of the fractional laser on the barrier function and structure of the stratum corneum (SC) and tight junctions (TJ) in inflamed skin.	Sampling methods: The study used in vitro skin models representing both healthy and disrupted barriers to investigate the effects of ablative (Er:YAG) and non-ablative (Er:glass) lasers on peptide and siRNA penetration. Time frame: Not explicitly mentioned.	Type: Experimental laboratory study. The study was conducted in a laboratory setting using in vitro skin models representing both healthy and disrupted barriers.	The fractional laser improved macromolecule permeation in barrier-disrupted skin but was less effective in diseased skin compared to normal skin.	The study's limitations include reliance on in vitro models, focus on only two skin conditions, lack of long-term evaluation, unaccounted variability in skin response, and limited applicability to other macromolecules and safety concerns.
LaBrasca et al. (2021) [11]	The study aimed to evaluate the effectiveness of high-peak power 1064 nm neodymium:yttrium aluminum garnet (Nd:YAG) laser treatment in managing refractory atopic dermatitis (AD). Specifically, the study intended to observe the long-term efficacy and effects of laser therapy on patients with biopsy-proven, poorly controlled, and long-standing atopic dermatitis.	Sampling methods: Not applicable; this is a case report featuring individual patient experiences. Time frame: The study spanned a 9-year period during which both patients underwent periodic laser treatments to manage their AD.	Type: Case report featuring two cases of patients with refractory atopic dermatitis who received long-term laser treatments over a 9-year period. The study was conducted in a clinical dermatological setting, focusing on the treatment of atopic dermatitis using high-peak power 1064 nm Nd:YAG laser therapy.	High-peak power 1064 nm Nd:YAG laser provided long-term symptom relief and disease control in refractory atopic dermatitis patients over nine years.	The primary limitation highlighted in the article is the small number of cases presented in this report. It is suggested that additional studies involving a larger number of patients are necessary to further understand the mechanisms and effectiveness of laser therapy for atopic dermatitis.
Park et al. (2013) [12]	The study aimed to evaluate the clinical efficacy and safety of 2790 nm erbium:yttrium scandium gallium garnet (Er:YSGG) laser therapy for reducing infraorbital dark circles in atopic dermatitis patients.	Sampling methods: Ten Korean subjects (eight women and two men) aged 21-46 years with mild atopic dermatitis were enrolled. Time frame: The study treatment was conducted with a 4-week interval between sessions. Assessments were performed at 2 months and 4 months following treatment.	Type: Uncontrolled and prospective pilot study. The study was conducted at Chung-Ang University Hospital in South Korea.	The 2790 nm Er:YSGG laser therapy improved symptoms by ~74% at 2 months and ~72% at 4 months, with high patient satisfaction and no severe side effects.	Small sample size: The study included a small number of patients. No control group: The study design lacked a control group.
Kim et al. (2021) [14]	The study aimed to evaluate the curative mechanism and optimal energy level of energy irradiation from a low-level laser (LLL) toward atopic dermatitis (AD) in a mouse model. Specifically, the objectives were to assess the effects of LLL irradiation on AD symptoms and cytokine levels, including interleukin (IL)-4, IL-6, tumor necrosis factor (TNF)-alpha, and interferon-gamma (IFN-gamma). Additionally, the study sought to examine the underlying mechanism of laser therapy's therapeutic effects and determine an optimal dosage.	Sampling methods: The study used 71 BALB/c mice divided into different groups: normal control (n=8), AD control (n=10), and four AD experimental groups with varying LLL irradiation levels (2 J/cm^2^, 4 J/cm^2^, 6 J/cm^2^, and 8 J/cm^2^). Time frame: The study applied LLL irradiation for 14 times over two weeks after AD induction.	Type: Randomized controlled trial using a mouse model. The study was conducted in the Center for Neurological Sciences at Sahmyook University in South Korea, utilizing laboratory animals.	LLL irradiation reduced serum IgE, cytokine levels, epidermal thickness, and mast cell counts, potentially alleviating AD symptoms and restoring tissue to normal.	The study's limitations include the use of a DNCB-induced atopy mouse model, which may not fully replicate human atopic dermatitis. The sample size may be limited, affecting statistical power. The study focused on short-term effects, lacking long-term evaluation of symptom progression and cytokine changes. Additionally, variations in individual immune responses and potential side effects of low-level laser therapy were not extensively analyzed. Further clinical studies are needed to confirm the findings in human subjects.
Martirosyan et al. (2022) [15]	To evaluate the impact of thymol administration and low-level laser therapy on changes in inflammatory and oxidative indicators, as well as lipid profiles in patients with type 2 diabetes. To investigate the effect of thymol oil extract on dermatitis in individuals with type 2 diabetes.	Sampling methods: The study involved 30 patients with type 2 diabetes and 30 healthy individuals as controls. The diabetic group was divided into four subgroups: untreated, treated with low-level laser, treated with thymol (25 mg/kg/30 days), and treated with thymol and laser. Time frame: Blood samples were collected before the study. Thymol gel oil extract (0.5%) was studied for its effect on reducing dermatitis in the feet of the diabetic group.	Type: Experimental study with a control group. No specific information about the setting where the study was conducted is mentioned.	Thymol combined with low-level laser therapy reduced cytokines (except IL-1α), cholesterol, triglycerides, AGEs, H₂O₂, MDA, and ox-LDL, but 0.5% thymol oil gel had no significant effect on dermatitis.	The study was limited by a small sample size, short duration, lack of a placebo control, and inconclusive effects of thymol on dermatitis.
Bhatia et al. (2015) [16]	The objective of the study was to evaluate the safety and efficacy of excimer laser therapy followed by narrowband UVB therapy for treating refractory exfoliative cheilitis in two female patients.	Sampling methods: Review of medical records of two female patients with refractory exfoliative cheilitis. Time frame: The study doesn't specify a set time frame but records the treatment and the subsequent follow-up over several months.	Type: Case report. The study was conducted in a dermatology clinic or a healthcare facility where patients were treated for their refractory exfoliative cheilitis.	Excimer laser therapy (600-700 mJ/cm², twice weekly) over several months significantly improved scaling, discoloration, and lip discomfort in both patients. Maintenance with a hand-held narrowband UVB device was effective post-treatment.	Limitations include small sample size and lack of standardization of starting dose and dose increments.
Leguina-Ruzzi et al. (2019) [17]	The objective was to assess the efficacy of polarized ultraviolet-free polychromatic light in managing non-atopic dermatitis, focusing on its impact on symptoms such as erythema, pruritus, and skin dehydration.	Sampling methods: Single case study -- a 67-year-old female patient suffering from moderate non-atopic dermatitis for 20 years. Time frame: The treatment duration was six weeks, involving daily light therapy applications for 10 minutes per affected area.	Type: Case report. The treatment took place in a clinical setting where the patient received daily light therapy for non-atopic dermatitis.	Polarized UV-free polychromatic light treatment significantly reduced erythema, pruritus, and skin dehydration without causing side effects or discomfort.	The case report explores the use of polarized UV-free polychromatic light therapy for non-atopic dermatitis but is limited by its single-patient design, lack of a control group, and potential conflict of interest due to an author's affiliation with the device manufacturer.
Oh et al. (2016) [19]	This study aimed to investigate the therapeutic effects of an excimer laser on pruritus and adhesion molecule expression in the skin of AD-induced NC/Nga mice, as well as the mechanisms underlying these effects.	Sampling methods: Six-week-old male NC/Nga mice were used as the study subjects. Time frame: The study spanned 4 weeks and involved multiple treatments and measurements over this period.	Type: experimental. The study was conducted in a controlled room maintained at specific temperature and humidity conditions with a 12-hour light-dark cycle.	In a mouse model, the 308 nm excimer laser effectively reduced AD-induced skin lesions, itching, and serum IgE. It suppressed key inflammatory cytokines and improved skin barrier function, highlighting its potential as a well-tolerated AD treatment.	While the findings demonstrated significant improvements in skin lesions, the study's limitations include its reliance on an animal model, which may not fully replicate human atopic dermatitis, and the absence of long-term safety and efficacy data. Additionally, the study did not explore potential side effects associated with the laser treatment.
Choi et al. (2016) [20]	The study evaluated the therapeutic effects of a newly developed gain-switched 311 nm Ti:Sapphire laser on atopic dermatitis (AD) using an NC/Nga mouse model. It compares clinical features, dermatitis severity, scratching behavior, serologic, and histological analyses between the Ti:Sapphire laser and conventional 311 nm narrowband-UVB treatment.	Sampling methods: A total of 50, six-week-old male NC/Nga mice were used. Time frame: The experiment involved a 28-day study period.	Type: Experimental study conducted on NC/Nga mice with induced atopic dermatitis.	The 311 nm Ti:Sapphire laser improved AD-like lesions and symptoms in NC/Nga mice by modulating immune responses, including hyper-IgE and Th2 cytokines, showing potential as targeted phototherapy for AD.	The inability to collect blood samples at the baseline (day 0) limited the comparison of the experimental group's results with those of the control group in the analysis of IgE and cytokines.
Yamazaki et al. (2021) [22]	The study aimed to retrospectively evaluate the efficacy and safety of femtosecond laser-assisted cataract surgery (FLACS) specifically for treating cataracts due to atopic dermatitis.	Sampling methods: This study involved a retrospective examination of 37 eyes from 30 atopic cataract patients. Time frame: The study spanned surgeries conducted from June 2012 to December 2016.	Type: Retrospective study. The study was conducted at the Omiya Nanasato Eye Institute, involving patients diagnosed with atopic cataracts, predominantly in Japan.	FLACS was successfully performed in atopic cataract patients, achieving free-floating capsulotomy in 86% of cases without radial capsular tears, vitreous loss, or lens dislocation.	The study evaluated femtosecond laser-assisted cataract surgery (FLACS) in patients with atopic dermatitis. Still, it was limited by its retrospective design and relatively small sample size, which may affect the generalizability of the findings.
Chu et al. (2017) [23]	Evaluate the efficacy of fractional CO_2_ laser treatment for lichen amyloidosis (LA) with atopic dermatitis (AD) in three cases. Report successful treatment outcomes of LA with underlying AD using fractional CO_2_ laser therapy.	Sampling methods: The study involved three patients in their thirties with chronic AD and accompanying LA. No randomization or control group was utilized. Time frame: The patients were treated on a monthly basis for two to three sessions. The follow-up period was more than one year.	Type: Case report. The study was conducted at a dermatology clinic, possibly affiliated with a medical institution.	Fractional CO_2_ laser effectively treated LA with underlying AD in three cases, improving symptoms after 2-3 cycles, though one patient experienced recurrence due to persistent scratching.	The study has limitations due to the small sample size (only three cases). The research lacked a larger controlled study to affirm the effectiveness of fractional CO_2_ laser in treating LA with underlying AD.
Dinish et al. (2023) [29]	Non-invasive biochemical analysis and comparison of atopic dermatitis and psoriasis skin using handheld confocal Raman spectroscopy	Sampling methods: Patients with atopic dermatitis (AD) and psoriasis. A total of 26 participants were included, comprising 11 healthy controls, 9 AD patients (4 severe, 4 moderate, and 1 mild), and 6 psoriasis patients (2 severe, 1 moderate, and 3 mild). Time frame: not specified.	The study was conducted at the National Skin Centre in Singapore, where patients with atopic dermatitis (AD) and psoriasis were recruited. The study also involved the inclusion of healthy controls for comparison. Data collection, including clinical assessments and physiological measurements, took place at this skin center.	The dual-wavelength handheld CRS system effectively analyzed biochemical components and skin physiology in AD and psoriasis. Raman spectra revealed distinct lipid-to-protein ratios, highlighting skin barrier dysfunction. AD showed higher epidermal water loss and reduced moisture, while psoriasis had higher moisture and ESR values, indicating stronger immune dysregulation. Ceramide and cholesterol levels varied between conditions, supporting the system’s potential for non-invasive diagnosis and treatment monitoring.	The study, a proof-of-concept with a small sample, assesses the handheld CRS system for AD and psoriasis but has limitations. It lacks demographic diversity, examines only two conditions, uses a cross-sectional design, and may not capture skin variability.
Krupka-Olek et al. (2022) [30]	To compare sensitization to inhaled allergens and immune profiles in patients with chronic AD, psoriasis, and healthy volunteers, while evaluating the influence of allergies on specific allergens and the severity of skin diseases.	Sampling methods: The study included patients aged 18 to 65 years diagnosed with either chronic atopic dermatitis (AD) or psoriasis (PS), exhibiting mild to severe forms. The study also included a control group of healthy volunteers without skin diseases, matching the age and sex distribution of the patient groups. Time frame: Patients were observed for at least 12 months.	The study was conducted at the Dermatology and Allergology Outpatient Clinic in Zabrze, affiliated with the Medical University of Silesia in Poland.	The study found higher inhaled allergen prevalence in AD than PS, with AD linked to environmental allergens. AD showed TH2-related cytokines (IL-4, IL-5, IL-6), while PS had elevated IL-17. Intrinsic AD shared molecular similarities with PS but required further study.	The study acknowledged limitations such as small sample sizes, especially in assessing allergies, and producing pilot results. The absence of tracking cytokine dynamics and limitations in the cytokine profile were noted, and a more extensive study to analyze disease severity and cytokine changes is recommended for future research.
Kamo et al. (2014) [31]	The antipruritic effects of excimer lamp irradiation on atopic dermatitis and its mechanism of action	Sampling methods: The study used 10-week-old male ICR and NC/Nga mice. Time frame: inducing dermatitis in NC/Nga mice over three weeks. Experimental procedures spanned several weeks, including dermatitis assessments, DRG neuron culture for 10 days, and excimer lamp irradiation at sub-erythema doses.	Study setting: Conducted at Juntendo University Graduate School of Medicine's experimental animal facility. Animal care: Followed NIH guidelines; mice maintained under a 12-hour light/dark cycle at 22–24°C with ad libitum food and water access.	Excimer lamp therapy reduced scratching and dermatitis in atopic-NC/Nga mice by inducing nerve fiber degeneration and lowering nerve density. It was safe, effective, and may be enhanced with emollients, though neuropathy risks require further study.	This studies' limitations include reliance on an animal model, which may not fully replicate human dry skin conditions, and a focus on short-term effects without assessing long-term outcomes or potential adverse effects of the treatment.
Kurosaki et al. (2020) [32]	To investigate the therapeutic effects of an excimer laser on pruritus and adhesion molecule expression in AD-induced NC/Nga mice and understand the mechanisms underlying these effects.	Sampling methods: The study used six-week-old male NC/Nga mice, inducing atopic dermatitis (AD)-like lesions with Dfb ointment. Mice were divided into treatment groups receiving NB-UVB or 308 nm excimer laser therapy. Time frame: Over four weeks, assessments included clinical severity, ear thickness, scratching behavior, inflammatory cytokines, TEWL, and histological analysis.	Type: Experimental animal study. Setting: Conducted in a controlled facility (24°C ± 2°C, 55% ± 15% humidity, 12-hour light-dark cycle) using NC/Nga mice.	The 308 nm excimer laser effectively improved AD symptoms in NC/Nga mice, reducing epidermal thickness, mast cell degranulation, scratching, IgE levels, and inflammatory cytokines. It offers advantages over traditional treatments like NB-UVB, disrupting the itch-scratch cycle and improving skin barrier function.	​The study on the effects of 308 nm excimer light treatment in atopic dermatitis patients has limitations, including a small sample size of 11 patients, which may limit the generalizability of the findings, and the absence of a control group, making it difficult to attribute observed changes solely to the treatment.
Hagiyama et al. (2013) [33]	To investigate the role of mast cell CADM1 in the pathogenesis of AD, CADM1 expression levels	Sampling method: atopic dermatitis (AD) was induced in mice using the hapten 2,4,6-trinitrochlorobenzene (TNCB). Mast cells were quantified histologically, and interactions between dorsal root ganglion (DRG) neurons and mast cells were examined using femtosecond laser-induced impulsive force loading and calcium imaging. Time frame: The specific time frame for these experimental procedures was not detailed in the provided information.	Setting: A laboratory study using BMMCs from BALB/c mice and IC2 mast cells cultured to express CADM1. Animal model: Conducted at Biostir Inc., Kobe, Japan, using a mouse model of atopic dermatitis (AD).	CADM1 upregulation in AD-like mast cells, regulated by MITF, enhances mast cell-sensory nerve interactions, contributing to the itch-scratch cycle. Its presence in skin cells may exacerbate pruritus, linking CADM1 to both neurological and psychological stress in AD.	The study's limitations include reliance on an animal model, which may not fully replicate human disease conditions, and a lack of long-term data on the effects of enhanced nerve-mast cell interactions.
Kobiela et al. (2023) [34]	How filaggrin levels are controlled before the formation of storing keratohyalin granules.	Sampling: Skin and blood samples were collected with ethical approvals; keratinocytes were isolated and cultured, including N/TERT-1 cells. Time frame: Duration not specified; experiments followed cell confluence and specific time intervals.	Type: Combination of experimental and observational study on excimer lamp effects in pruritic AD model mice and DRG neurons. Setting: Controlled laboratory environment using skin and blood samples from donors and patients, with keratinocyte isolation and molecular experiments.	Filaggrin plays a key role in epidermal barrier formation, keratohyalin granule formation, and immune regulation. The study identifies a mechanism for controlling intracellular filaggrin levels via exosomal export, which may impact systemic immunity. *Staphylococcus aureus* may disrupt this process, contributing to AD barrier dysfunction through TLR2 signaling. Targeting this pathway could offer therapeutic potential for AD.	Limitations include the reliance on in vitro keratinocyte cultures, which may not fully replicate the complex in vivo skin environment, and the lack of clinical data to confirm these findings in patients with atopic dermatitis.
Liew et al. (2020) [35]	Investigates aberrant microRNA (miRNA) expression as a cause of barrier defects in atopic dermatitis (AD). Explores the molecular mechanisms of the dysregulated miRNA network. Identifies potential drugs that modulate miRNA expression to repair the defective skin barrier in AD.	Sampling methods: The study involves the use of cell lines such as N/TERT-1 keratinocytes and HEK293T cells for in vitro experiments. Additionally, human skin biopsy specimens were obtained from 10 patients with atopic dermatitis (AD) and 6 healthy individuals for the ex vivo human skin organ culture model. Time frame: The specific time frame for the study is not mentioned in the provided text, but various time points are mentioned within the study methods, such as 48 hours after transfection for cell cultures.	Setting: Controlled laboratory environment. Method: Human skin organ culture using skin explants from elective surgery patients.	The study highlights miR-335’s role in epidermal homeostasis and its loss in AD, leading to barrier defects. MiR-335 regulates keratinocyte differentiation by suppressing SOX6, which, when overexpressed in AD, inhibits differentiation via chromatin remodeling proteins. Histone deacetylases (HDACs) regulate miR-335, and HDAC inhibitors like belinostat show potential in restoring skin barrier function, offering a promising AD treatment approach.	Limitations include reliance on in vitro and ex vivo models, which may not fully replicate in vivo conditions, and the absence of clinical trials to confirm belinostat's efficacy and safety in human patients.
Lu et al. (2018) [36]	To investigate the key signaling molecules reflecting the progression of inflammation in atopic dermatitis.	Sampling methods: The study recruited a total of 11 patients with moderate to severe atopic dermatitis and 10 normal control subjects. Patients with AD were selected based on standard criteria and guidelines for diagnosing atopic dermatitis. The study does not provide detailed information on the specific criteria used for the selection of patients or controls. Time frame: The text does not specify the exact time frame during which the study was conducted. It does mention that the protocol was approved by the Ethics Committee of the Third XiangYa Hospital, Central South University.	Type: Observational case-control study. Setting: Conducted at Third XiangYa Hospital, Central South University, comparing immunohistochemical markers in AD patients and controls.	TGF-β signaling in AD has dual roles, promoting immune infiltration and fibrosis while also suppressing certain immune cells. Enhanced TGF-β signaling upregulates TGF-βR1, activating pathways like p-Smad2, JNK, PKC-βII/ζ, and c-Src, leading to inflammation and fibrosis. RhoA activation further drives immune cell infiltration. TGF-βR1, JNK, and c-Src are potential therapeutic targets for AD treatment.	The study's limitations include a small sample size and the lack of functional assays to elucidate the specific roles of these signaling pathways in the pathogenesis of atopic dermatitis.
Segaud et al. (2022) [37]	To Investigate the impact of allergen exposure on cytokine microenvironments. Examines the roles of TSLP and IL-1 in allergen sensitization and allergic asthma development.	The study used reporter mice. The study employed laser-assisted skin microporation (LMP) for cutaneous sensitization with house dust mite (HDM) allergen, intranasal HDM challenge, antibody administration, bronchoalveolar lavage cell analysis, airway responsiveness to methacholine testing, quantitative RT-PCR, serum immunoglobulin determination, protein level determination, cell preparation for flow cytometry analyses, flow cytometry analyses, histopathology, immunohistochemistry, and RNAscope in situ hybridization.	Type: Experimental study. Setting: Mice housed in controlled conditions (22°C, 40-60% humidity, 12-hour light/dark cycle) with unrestricted food and water access.	Deeper dermacutaneous (d.c.) sensitization triggers stronger allergic and lung inflammation than epicutaneous (e.c.) sensitization. TSLP drives e.c. sensitization, while IL-1β promotes d.c. sensitization independently of TSLP. Neutrophils play a role in allergic sensitization and asthma. Targeting both TSLP and IL-1β pathways could benefit moderate-to-severe AD patients.	The study found that IL-1β blockade with Anakinra had limited efficacy in reducing asthma symptoms, possibly due to genetic factors. It calls for further research into IL-1β's role in Th2/Tfh differentiation, neutrophils in skin sensitization, and corticosteroid resistance in dermacutaneous-induced asthma.
Luger et al. (2021) [38]	Propose a treatment algorithm for patients with mild-to-moderate and severe atopic dermatitis flares in daily clinical practice.	Sampling methods: The panel consisted of 15 dermatology and allergy experts from eight different countries. They developed the algorithm based on a review of published treatment guidelines, their clinical experience, and an evaluation of relevant literature. Time frame: The algorithm was developed based on literature published up to May 2018.	Type: Expert consensus study. Scope: Developed a practical AD treatment algorithm with input from experts across eight countries, later adapted for the Middle East and Asia.	The AD management algorithm emphasizes early severity assessment with SCORAD, early intervention with emollients, and patient education. For mild-to-moderate AD, TCIs like pimecrolimus (preferred for sensitive areas) and tacrolimus are recommended, while severe flares may require initial TCS use. Maintenance therapy focuses on flare prevention and reducing TCS reliance. Novel topical treatments show promise but may face cost limitations. The goal is to lessen AD’s impact on patients and healthcare systems.	​The proposed treatment algorithm is based on expert consensus rather than empirical clinical trial data, potentially limiting its generalizability across diverse patient populations.
Luger et al. (2020) [39]	Provides an overview of the presentations and discussions from the fourth successful IDeA meeting, summarizing the key insights shared by dermatologists from across the globe.	The article is a review based on discussions from the IDeA 2019 conference, summarizing expert insights on dermatological advancements and challenges without a traditional study setting or participant enrollment.	Type: overview	The study explores AD diagnosis, emphasizing subjective criteria, immune pathways, microbiome influences, and patient stratification while identifying four Th2-driven endotypes and the microbiome's diagnostic potential. It highlights the importance of early and prolonged treatment, preferring TCIs over corticosteroids, promoting holistic care, and reviewing advanced therapies like monoclonal antibodies, JAK inhibitors, and PDE-4 inhibitors for symptom management.	The article provides an update on developments and challenges in dermatology based on discussions from the Interactive Derma Academy (IDeA) 2019. However, as a conference report, it may not encompass all recent advancements in dermatology, and the insights presented are primarily based on expert opinions rather than empirical data.
Szegedi et al. (2015) [40]	To investigate the cytokine milieu in interstitial fluid (ISF) collected from chronic lesional AD skin as compared to ISF from non-lesional AD skin and/or healthy donor skin.	Sampling: Included AD patients (meeting Millennium Criteria) and healthy volunteers. Collected blood, skin biopsies, and interstitial fluid via punch biopsies for cytokine and PBMC analysis. Time frame: Conducted in two periods: September-October 2009 and May-June 2011.	Type: observational study, cross-sectional study. The study was conducted in a clinical setting and involved participants with AD and healthy volunteers.	The study analyzed ISF from chronic AD skin, identifying elevated IP-10, MIG, IL-1β, IL-18, IL-5, and IL-13, which correlate with AD severity and reinforce Th2-driven inflammation. FLG-null mutations were linked to more severe cases, while G-CSF levels were lower in AD skin. Additionally, some cytokines and chemokines were undetectable, providing further insights into the inflammatory mechanisms and immune dysregulation underlying AD pathogenesis.	Limitations include the relatively small sample size of 16 AD patients and 12 healthy individuals, which may affect the generalizability of the findings. Additionally, the study's cross-sectional design captures cytokine levels at a single time point, limiting insights into dynamic changes over time.
Try et al. (2023) [41]	The study evaluates polymeric nanoparticles for targeted drug delivery in atopic dermatitis (AD) lesions, minimizing exposure to healthy skin. It examines nanoparticle penetration, barrier differences, and tight-junction protein changes, concluding their potential as a selective drug delivery tool for inflamed skin.	Sampling: Ten healthy volunteers and six AD patients recruited from a dermatology department based on specific criteria. Time frame: Three-day clinical study with topical application and biopsies; porcine model examined on days 1, 3, and 10 post-application.	Type: Prospective clinical investigation (proof-of-concept). Settings: Conducted in a hospital dermatology department (patient recruitment, nanoparticle application), laboratory (nanoparticle synthesis and analysis), and animal research facility (porcine AD model, confocal microscopy).	Polymeric nanoparticles accumulated more in AD lesions than in healthy or non-lesional skin, with enhanced retention in the thickened epidermis. Reduced barrier proteins (Claudin-1, Claudin-4, ZO-1) facilitated deeper penetration, supported by animal models. These nanoparticles show potential for targeted drug delivery, minimizing systemic absorption and improving treatment for chronic skin diseases like AD, psoriasis, and pemphigus.	Limitations include a small sample size and the lack of long-term safety evaluations, which may affect the generalizability and comprehensive assessment of the findings.
Lee et al. (2022) [42]	The study investigates the use of picosecond- and nanosecond-domain Nd:YAG lasers to enhance peptide delivery with minimal skin invasiveness. It compares peptide absorption across different laser types, assesses skin structure changes, and evaluates in vitro and in vivo effects on barrier-deficient and inflamed skin.	Sampling methods: No human subjects involved. In vitro experiments on excised pig skin. In vivo experiments on crossbred pigs, Balb/c mice, and nude mice. Time frame: The specific time frame for the study is not mentioned, but various experiments were conducted at different time points, as needed.	Type: Experimental laboratory and animal study. Laboratory settings for in vitro experiments and animal facilities for in vivo experiments.	The study found that non-ablative picosecond- and nanosecond-domain Nd:YAG lasers enhance topical peptide delivery with minimal skin damage and faster barrier recovery. Peptide absorption varied by lipophilicity and size, with copper tripeptide (CT-1) showing better penetration. Nanosecond-domain Nd:YAG lasers were particularly effective for follicular delivery, offering a safe and efficient method for cosmeceutical applications.	Limitations include the use of pig and mouse skin models, which may not fully replicate human skin physiology, and the lack of long-term safety data regarding repeated laser exposure.

Characteristics of AD

AD and psoriasis displayed similar symptoms, but they were distinct diseases. Comparisons between AD and psoriasis patients enhanced the understanding of AD pathogenesis. Skin conditions in a total of 26 participants, including nine AD patients, six psoriasis patients, and 11 healthy controls, were observed and compared by the Domain Specific Review Board of the National Healthcare Group, Singapore. Non-invasive Raman spectroscopy was used to identify similarities and differences between AD and psoriasis. Both AD and psoriasis skin differed from healthy skin in terms of C-C stretching vibration, CH_2_ stretching, and protein bonds. A reduced lipid-to-protein ratio observed in Raman peaks indicated that AD and psoriasis had an underlying skin barrier dysfunction. The unique characteristics of AD that Raman spectroscopy identified were reduced skin moisture, increased epidermal water loss, and less acidic pH than healthy skin. However, cholesterol levels were not altered. Non-lesional AD had higher ceramide (CER 2 and CER 3) levels than lesional AD.

Allergens and immune profiles of AD and psoriasis patients aged 18-65 years were observed for at least one year in the study by Krupka-Olek et al. [30]. AD patients had a higher prevalence of inhaled allergen allergies than psoriasis patients. The mite allergens played an essential role in AD allergy, while dust allergy did not manifest in the severe course of AD. Higher concentrations of IL-4, IL-5, and IL-6 were found in AD patients, thus confirming the TH2-polarized nature of AD.

Laser Treatment

Several studies using NC/Nga mice models evaluated the effectiveness of excimer lamp irradiation as a treatment for AD. Kamo et al. [31] found that excimer lamp irradiation decreased scratching behaviors in dermatitis mice, caused nerve fiber degeneration, and reduced epidermal and intraepidermal nerve fiber density and Nmnat2. Additionally, an excimer lamp with a cutoff filter was deemed safe and effective in decreasing keratinocyte DNA damage. Later, it was found that the serum levels of total IgE and multiple inflammatory cytokines, such as IL-1α, IL-1β, IL-6, MCP-1, and IL-31, were suppressed by the 308 nm excimer laser. In 2020, the 308 nm excimer laser was discovered to effectively reduce mast cell degranulation and epidermal thickness in dermatitis mice.

Picosecond- and nanosecond-domain Nd:YAG laser, another laser treatment, was evaluated for its effectiveness in enhancing cosmeceutical peptides delivery. Pig and mice models indicated that picosecond- and nanosecond-domain Nd:YAG laser facilitated topical peptide permeation to a deeper level compared to microsecond-domain ablative lasers, and skin barrier function recovered faster than with ablative lasers.

Potential Therapeutic Targets

Many studies investigated the molecular mechanisms of AD and identified potential therapeutic targets. AD-like lesional mast cells had an elevated CADM1 expression. This upregulation of CADM1 facilitated mast cell adhesion and communication with sensory nerve cells, contributing to AD's itch-scratch cycle. Various skin cell types contained CADM1, which explained the expanding pruritus of AD.

Filaggrin was essential in programmed keratinocyte death to help stratum corneum formation, and the export of profilaggrin-derived products via small extracellular vesicles (sEVs) or exosomes was essential for keratinocyte survival. Since filaggrin suppressed innate immunity pathways and antigen-specific T-cell responses, filaggrin-containing exosomes impacted immune responses when diffused to peripheral tissues. Filaggrin loading into sEV/exosomes required TLR2 signaling, which was linked to innate recognition of *Staphylococcus aureus*. Because *S. aureus* worsens AD, the mechanism of profilaggrin removal via sEV/exosomes was identified as a potential treatment for AD.

The microRNA-335 (miR-335) was abundant in healthy epidermis but suppressed in AD lesional skin. The suppression of miR-335 elevated SOX6 in AD, and SOX6 suppressed epidermis differentiation and cornification, which initiated AD through allergen sensitization and antigen penetration. The histone deacetylases (HDACs) were considered a potential treatment for AD because HDAC1 and HDAC2 were found to be abundant in the miR-335 promoter region and elevated differentiation-related markers in AD. Clinical trials for belinostat, an effective HDACI, were ongoing.

AD skin had an unregulated TGFβR1 and downstream signaling components like JNK and c-Src. RhoA activation induced by TGFβ increased AD's immune cell infiltration and skin fibrosis. Therefore, AD could be alleviated by suppressing TGFβR1, JNK, and c-Src.

Practical Algorithm

The practical algorithm was updated based on new AD discoveries. Updated to 2021, it was recommended that AD should be assessed with the SCORAD tool and early intervention with emollients. Topical calcineurin inhibitors (TCIs) like pimecrolimus (for sensitive areas) and tacrolimus were recommended for mild-to-moderate AD (including infants). For severe AD flares in sensitive areas, TCS were recommended initially, but the main goal was to reduce flares and lower TCS use.

Furthermore, two studies focused on analyzing stepwise procedures for treating AD, including the addition of novel treatments like laser therapy. It was important to remember that AD varied from person to person, and the effectiveness of treatments also differed. Treatment plans needed to be tailored to an individual's needs and might have required adjustments over time. Consulting with a dermatologist or healthcare provider for a proper diagnosis and personalized treatment plan was imperative. By implementing some of the discussed methods, more research could be conducted on the effectiveness of laser therapies for treating AD.

Discussion

This scoping review aimed to comprehensively assess the efficacy and suitability of phototherapy as a treatment modality for AD. The review focused on empirical studies published within the past decade, with key objectives including examining patient diversity, defining phototherapy usage, evaluating treatment outcomes, and integrating findings from various studies.

Scoping reviews provided insights into the current evidence on a specific topic. While their methodology closely resembled that of systematic reviews, they did not produce critically appraised and synthesized results. Consequently, they did not necessitate an assessment of methodological limitations or risk of bias [24].

The primary research question of this review was whether laser therapy could effectively treat and prevent the recurrence of AD. Two sub-questions explored whether specific laser therapies were more effective for long-term AD management and whether their efficacy varied across different patient populations. A total of 27 studies were selected to address these questions, with findings indicating that two laser therapies demonstrated efficacy in the long-term management of AD [4,38,39].

Effectiveness of Laser Therapies

Phototherapy, particularly excimer laser therapy, has been explored as a treatment option for AD due to its ability to target inflammatory pathways and modulate the immune response. The 308 nm excimer laser had been widely studied for its efficacy in reducing scratching behaviors, inducing nerve fiber degeneration, and suppressing inflammatory cytokines, all of which were crucial for mitigating the chronic symptoms of AD [19,31,40-42]. Clinical trials had also demonstrated that the excimer laser could alleviate pruritus and reduce disease severity, with significant improvements observed in patients with moderate-to-severe AD [18]. Furthermore, studies suggested that excimer laser therapy might have influenced the skin microbiome, potentially reducing pathogenic bacterial colonization, which was often exacerbated in AD patients [33].

In addition to the excimer laser, the picosecond- and nanosecond-domain Nd:YAG laser has been identified as a potential therapy for AD due to its role in enhancing topical peptide permeation and accelerating skin barrier recovery [32]. The 1064 nm Nd:YAG laser had also been successfully used to treat refractory AD cases, demonstrating reductions in inflammation and skin thickening [11,42]. However, while these laser technologies showed promise, they had yet to be widely tested on diverse AD patient populations, limiting the understanding of their broader efficacy.

Molecular Insights and Emerging Therapies

At a molecular level, AD differed significantly from psoriasis, as it was characterized by reduced skin moisture, increased epidermal water loss, and a less acidic pH, all of which contributed to an impaired skin barrier [29]. The TH2-polarized immune response in AD had been well-documented, with elevated levels of cytokines such as IL-4, IL-13, and TSLP, as well as a strong association with mite allergens [30,40]. Cytokine profiles in the interstitial fluid of chronic AD skin further supported this inflammatory process, with significant elevations in inflammatory mediators such as IL-31, a key driver of pruritus [40]. Given the role of these cytokines in AD pathogenesis, therapies targeting these molecular pathways, including phototherapy, hold significant therapeutic potential.

Several molecular therapeutic targets have been identified in AD, including CADM1, filaggrin, HDAC, TGFβR1, JNK, c-Src, IL-1β, and TSLP [19,34-38]. Recent findings suggested that the 308 nm excimer laser could modulate immune responses by downregulating inflammatory markers, potentially influencing these therapeutic targets [19,31]. However, additional research was needed to determine whether laser therapy could effectively alter cytokine expression and improve long-term disease outcomes.

The Role of Polymeric Nanoparticles in AD Therapy

An emerging area of interest in AD treatment was the use of polymeric nanoparticles for targeted drug delivery and skin barrier repair. Polymeric nanoparticles enhanced drug stability, bioavailability, and penetration through the compromised AD skin barrier, making them an attractive option for improving the efficacy of topical therapies [41]. Studies have shown that polymeric nanoparticles accumulate differently in healthy, non-lesional, and lesional AD skin, indicating their potential for precise, localized therapy that targets inflamed skin while minimizing systemic side effects [41].

Given that fractional laser therapy had been shown to enhance cutaneous macromolecule delivery, there was potential for integrating nanoparticle-based drug carriers with phototherapy to optimize AD treatment [10]. This approach might have improved treatment penetration and efficacy while reducing the need for systemic immunosuppressants, which carry the risk of adverse effects [7]. Future studies should explore how polymeric nanoparticle formulations interact with laser therapy to determine their synergistic potential in AD management.

Limitations of the Studies in the Review

One major limitation of the reviewed studies was the small sample size. Throughout the review process, challenges arose in finding sufficient research on the efficacy of laser therapy for AD. Many studies focused on other dermatological conditions, such as psoriasis and vitiligo, making it difficult to draw definitive conclusions about the efficacy of laser therapy specifically for AD [8,9]. This restriction resulted in a relatively small sample pool for this review.

Another limitation was the variability in study designs, with some studies lacking control groups or long-term follow-ups. The heterogeneity in treatment protocols, including differences in laser wavelengths, energy settings, and exposure durations, complicated direct comparisons between studies [13]. Additionally, most of the existing research had been conducted in specific populations, often excluding racial and ethnic diversity, which might have limited the generalizability of findings to broader patient groups [5].

Limitations of the Review Process

Several factors might have influenced the review process. First, selection bias may have occurred, as each research team member participated in the article selection phase, increasing the possibility that relevant studies were overlooked. Additionally, the literature search was limited to selected databases, which may have resulted in the omission of relevant studies indexed in other databases or unpublished sources. Studies also originated from different countries with varying healthcare systems, which might have impacted the applicability of findings to the U.S. and other Western healthcare contexts [4]. Finally, as laser therapy is a relatively novel treatment approach for AD, new studies with potentially conflicting or complementary findings may have been published after this review was conducted.

Implications for Future Research

Although the 308 nm excimer laser and picosecond- and nanosecond-domain Nd:YAG laser had demonstrated efficacy in treating AD, further research was needed to explore their effects across diverse patient populations. Additionally, studies should investigate the relationship between topical peptide permeation and AD pathogenesis to confirm whether Nd: YAG laser therapy directly benefits AD management [32].

Future research should also assess the impact of laser therapy on molecular therapeutic targets, particularly whether laser-induced cytokine modulation could improve long-term treatment outcomes [19,31]. Moreover, the combination of laser therapy with polymeric nanoparticle-based drug delivery warranted further exploration, as this approach could enhance therapeutic precision and effectiveness [41].

Longitudinal studies evaluating laser therapy efficacy in AD patients of different races, sexes, and ages would be essential in determining whether phototherapy could serve as a reliable long-term treatment for AD [5]. Given the promising findings from existing studies, integrating laser therapy with molecular and nanotechnology-based approaches might represent the future of AD management.

## Conclusions

Due to the commonality and chronicity of AD, efficacious treatments to halt flare-ups and prevent reoccurrences were necessary. Laser therapy appeared to be a promising treatment for the persistent and life-impacting condition of AD. This scoping review assessed the efficacy of laser therapy in patients with AD by evaluating 27 publications, including original research studies, treatment guidelines, case reports, and review articles. Different phototherapy methods, including the Nd:YAG and 308 nm excimer lasers, demonstrated laser therapy’s effectiveness in suppressing inflammatory cytokines in patients with AD. Additionally, this review revealed the specific molecular targets of AD phototherapy treatments, indicating the precise nature of laser therapy in suppressing particular biomarkers. This precision might have pointed to the potential of phototherapy to cure AD, comparable to the efficacy of chemotherapy treatments in silencing biomarkers in patients with cancer. However, this efficacy remained speculative due to the hypothetical nature of the studies on the molecular analysis of phototherapy.

This scoping review was unable to determine the long-term effectiveness of phototherapy for treating AD, nor the specific populations of AD patients in which phototherapy was effective, due to the lack of current research on the topic. Gaps in knowledge existed between the hypothetical use of phototherapy and its actual use in clinical practice. Future research on the utilization of phototherapy to treat and prevent the re-emergence of AD was needed to better identify specific treatment methods and protocols for laser therapeutics used in patients with AD. Additionally, further research should have focused on the ability of laser treatment for AD to manage symptoms, reduce inflammation, and improve the overall quality of life in a wide variety of populations.
